# Insulin Sensitivity Assessed by Stable Isotopes with Oral Glucose Administration: Validation with Euglycaemic Clamp

**DOI:** 10.1155/2013/189412

**Published:** 2013-11-03

**Authors:** Leslie Bluck, Rachel Williams, Sarah Jackson, Burak Salgin, Carlo Acerini, David Dunger

**Affiliations:** ^1^MRC-Human Nutrition Research, 120 Fulbourn Road, Cambridge CB1 9NL, UK; ^2^Department of Paediatrics, Cambridge University, Addenbrooke's Hospital, Hills Road, Cambridge CB2 0QQ, UK

## Abstract

Methods of determining insulin sensitivity that use an oral challenge of glucose are preferred to those using intravenous administration since the measurement is made in conditions more akin to normal physiology. One previously reported protocol (ODILE) studies glucose uptake in isolation from absorption and endogenous production by the intravenous administration of tracer approximately forty-five minutes after the oral dose is given. However, this methodology has not been validated against other accredited procedures. This study utilizes the euglycemic hyperinsulinemic clamp in order to validate the ODILE method.

## 1. Introduction

The determination of the parameters of glucose metabolism, and in particular peripheral insulin sensitivity, is of great interest to clinicians and researchers interested in the aetiology of type 2 diabetes and the metabolic syndrome. Currently, there are two procedures accredited with providing estimates of peripheral insulin sensitivity on a cardinal scale [1]: the euglycaemic clamp [2] and the intravenous glucose tolerance test (IVGTT) interpreted via the minimal model [3]. The clamp methodology is clinically intensive and usually applicable only to studies with relatively few subjects (*n* < 20). The IVGTT is more widely employed and has been used in intervention studies with many hundreds of participants [4, 5]. The IVGTT, however, fails to conform to Groop's [6] list of desirable properties of a methodology to measure insulin sensitivity, inasmuch as it creates unphysiological conditions. Intravenous administration of a glucose bolus gives an almost instantaneous increase in plasma glucose from basal (fasting) levels to a condition where the renal threshold is frequently exceeded, and because of the transitory nature of the glucose input, the period of hyperglycemia is generally less than one hour in normal subjects. During this time, the concentration of glucose in the peripheral circulation exceeds that in the portal vein, in contrast to normal post-prandial conditions. As a consequence, only first phase insulin secretion can be reliably determined, yet such a response never occurs in isolation in the normal state. Secondly, in the absence of incretin effects, the endogenous insulin response is too small (~200 pM) and too transitory to be a satisfactory stimulus for peripheral glucose uptake. Although the latter can be ameliorated to some extent by exogenous insulin administration [7], this removes the physiological conditions of the test even further from reality.

These objections could be overcome if oral administration of the glucose load is incorporated into the test. The major drawback of this approach when compared with the IVGTT is that the rate of appearance of glucose in the blood is unknown with oral dosing, and separation of the kinetics of appearance and disappearance is not possible without additional information or assumptions. The incorporation of a small bolus dose of labeled glucose near to the maximum hyperglycaemia has been proposed as a method of overcoming this [8]. A report of proof of principle demonstrated that such a test gave repeatable estimates of peripheral insulin sensitivity but was unable to validate the new test (originally called the OSIVGTT, but more recently renamed ODILE for Oral Dose Intravenous Label Experiment) by comparison with an established technique. Here, we report such a validation using a stable-labeled euglycaemic clamp as the reference method.

## 2. Materials and Methods

### 2.1. Subjects

Twelve healthy nonobese volunteers aged 18–35 years with body mass index in the range 20–30 kgm^−2^ were recruited in the study by advertisement. Written informed consent was obtained from each subject. The study was approved by Cambridge Local Research Ethical Committee. All the volunteer work took place in the Wellcome Trust Clinical Research Facility, Addenbrooke's Hospital, Cambridge. Each volunteer underwent a hyperinsulinaemic euglycaemic clamp and an Oral Dosing Intravenous Labeled Experiment (ODILE) in random order within 15 days of each other.

### 2.2. Test Protocols

#### 2.2.1. Hyperinsulinaemic Euglycaemic Clamp

Subjects were admitted to the ward at 07:00 h following an overnight fast. Blood sampling via an indwelling cannula began at 07:45 h. and continued for 5.25 h. Three basal blood samples were drawn between 07:45 and 08:00 h., and then a single-step hyperinsulaemic euglycaemic clamp study was performed. A primed (930 *μ*mole) infusion (9.3 *μ*mole·min^−1^) of [6,6]-^2^H_2_-glucose was administered throughout the clamp procedure to determine the degree of suppression of endogenous glucose production. A 2.5 h  stabilization period was allowed during which two further blood samples were taken at 09:00 and 10:00 h, before the normoinsulnemia study phase of the clamp was begun, where seven blood samples were taken at five minute intervals, starting at 10:30 h. At the end of this sampling period, a primed (17.5 pmole·kg^−1^) infusion (3.5 pmole·min^−1^)·kg^−1^) of insulin was begun and continued until the end of the clamp study. The insulin dose was chosen to raise plasma insulin concentrations by ~200 pmole·L^−1^, while the serum glucose remained at the pretest level (~4 mmole·L^−1^). During the period of hyperinsulinaemia, blood glucose was determined every five minutes, and maintained at the mean level determined during the normoinsulinemia phase by a variable rate infusion of 20% glucose solution enriched with 0.7% of the [6, 6]-^2^H_2_-glucose tracer. After a stabilization period of 1.5 h, seven blood samples were taken, one every five minutes, to characterize the hyperinsulinaemic phase, and complete the clamp study. The infusions were then terminated, the cannulae were removed, and the subject was given breakfast.

Serum glucose concentrations were measured using a model 2300 STAT Plus Glucose and Lactate analyzer (YSI, Yellow Springs, Ohio, USA), which uses whole blood and has a precision of ±0.2 mmol/L at normal fasting levels. Plasma insulin concentration was measured using ELISA (DSL, Webster, Texas, USA) according to the manufacturer's instructions.

Isotopic composition of the glucose was determined using the *α*-D-glucofuranose cyclic 1,2 : 3,5-bis (methyl boronate)-6-trifluoroacetate derivative [9] using a 5973 mass selective detector (Agilent Technologies, Palo Alto, California, USA). The spectral region at 240–242 Th was monitored, and the isotopic composition of the glucose was determined by using a fitting algorithm [10].

#### 2.2.2. ODILE

The protocol for the ODILE test has been described previously [8]. The procedure can be described as a standard oral glucose tolerance test, modified by the addition of an intravenous bolus of 1.38 mmole (250 mg) [1]-^13^C-glucose administered 45 minutes after the oral glucose has been taken. Blood samples are taken at −15, −10, −1, 15, 30, 45, 46, 47, 48, 49, 50, 51, 53, 55, 57, 59, 61, 63, 65, 70, 75, 80, 85, 90, 95, 100, 105, 115, 125, 135, 150, 165, 180, and 225 minutes after oral dosing.

Glucose and Insulin measurements were made as for the clamp. ^13^C isotopic composition of the glucose was determined by GC/C/IRMS on an Isoprime instrument (Micromass UK Ltd., Manchester, England) with a CuO packed combustion furnace operating at 850°C. The ratio of the 44 and 45 Th isotopologues of the generated CO_2_ was determined using the method of Bluck and Coward [11], and these were converted [12, 13] to tracer/tracee ratios using a derived value for pure tracer of 8991‰  for the glucose derivative used in this work.

**Figure 1:**
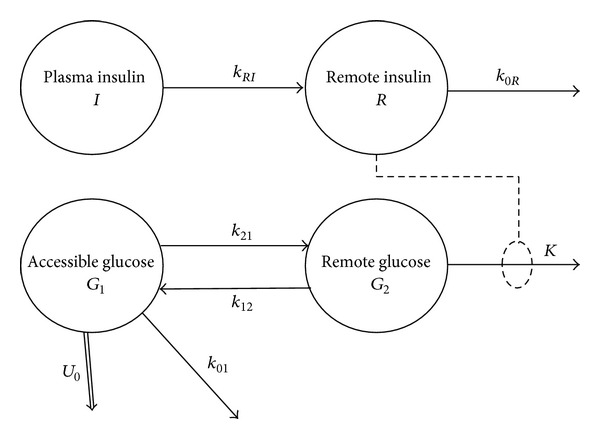
The two-compartment minimal model.

The data obtained were interpreted by the two-compartment minimal model [14–16] as implemented by Bluck et al. [8]. The structure of this model is shown in Figure 1. Glucose is described as existing in two freely interchanging compartments, with insulin independent uptake occurring from the accessible pool by two routes. The first of these corresponds to saturated GLUT2 transport and is represented by a constant flux, whilst the second describes nonsaturated transport via GLUT1 and GLUT3, and is therefore first order (linear) in the quantity of glucose in the pool. Insulin assisted (GLUT4) uptake is assumed to occur only from the inaccessible pool. In common with the other minimal models, insulin is also assumed to be distributed between two pools, acting from the inaccessible (remote) one.

The model is specified by three differential equations:

(1) $$\begin{array}{c} \frac{dR}{dt}=k_{RI}V_{I}\left[I\right]-k_{0R}R,\\ V_{1}\frac{d\left[G_{1}\right]}{dt}=H-U_{0}-\left(k_{01}+k_{21}\right)V_{1}\left[G_{1}\right]+k_{12}G_{2},\\ \frac{dG_{2}}{dt}=k_{21}V_{1}\left[G_{1}\right]-k_{12}G_{2}-KRG_{2}, \end{array}$$

*U*_0_ is a term denoting a constant (time-independent) contribution to glucose uptake, and *H* is endogenous glucose production. Note that there has been considerable care to maintain the distinction between the concentration of glucose in the accessible pool, [*G*_1_], and the total quantity in the remote pool, *G*_2_.

In common with all such models, reparameterization to obtain an expression for insulin action is desirable: in this case we adopt that proposed by Mari [17].

(2) $$\begin{array}{c} Z=\frac{k_{0R}}{k_{RI}V_{I}}R. \end{array}$$

Which leads to the equivalent set of equations:

(3) $$\begin{array}{c} \frac{dZ}{dt}=k_{0R}\left(\left[I\right]-Z\right),\\ V_{1}\frac{d\left[G_{1}\right]}{dt}=H-U_{0}-\left(k_{01}+k_{21}\right)V_{1}\left[G_{1}\right]+k_{12}G_{2},\\ \frac{dG_{2}}{dt}=k_{21}V_{1}\left[G_{1}\right]-k_{12}G_{2}-\kappa ZG_{2}, \end{array}$$

where

(4) $$\begin{array}{c} \kappa =\frac{Kk_{RI}V_{I}}{k_{0R}}. \end{array}$$

It can be shown [14] that this model is not identifiable by a labeled bolus delivered to the accessible glucose pool, and, therefore, two internal constraints are required. These require consideration of the basal (equilibrium) state, from which it is derived that

(5) $$\begin{array}{c} Z_{b}={\left[I\right]}_{b},\\ G_{2b}=\frac{k_{21}V_{1}{\left[G_{1}\right]}_{b}}{k_{12}+\kappa {\left[I\right]}_{b}}. \end{array}$$

The proposed internal constraints [15] give further relationships between the parameters:

(6) $$\begin{array}{c} U_{0}=\alpha \left(1+\beta \right)\frac{k_{21}\kappa {\left[I\right]}_{b}V_{1}{\left[G_{1}\right]}_{b}}{k_{12}+\kappa {\left[I\right]}_{b}},\\ k_{01}=\left\{\alpha -\beta \left(1-\alpha \right)\right\}\frac{k_{21}\kappa {\left[I\right]}_{b}}{k_{12}+\kappa {\left[I\right]}_{b}}, \end{array}$$

where *α* and *β* have fixed values of 0.465 and 3, respectively. The kinetics for the labeled material injected during the test are then described by

(7) $$\begin{array}{c} \frac{dZ}{dt}=k_{0R}\left(\left[I\right]-Z\right),\\ V_{1}\frac{d\left[G_{1}^{*}\right]}{dt}=-U_{0}\frac{\left[G_{1}^{*}\right]}{\left[G_{1}\right]}-\left(k_{01}+k_{21}\right)V_{1}\left[G_{1}^{*}\right]+k_{12}G_{2}^{*},\\ \frac{dG_{2}^{*}}{dt}=k_{21}V_{1}\left[G_{1}^{*}\right]-k_{12}G_{2}^{*}-\kappa ZG_{2}^{*}. \end{array}$$

The kinetics are therefore described in terms of five unknown macroparameters, *V*_1_, *k*_21_, *k*_12_, *k*_0*R*_, and *κ*. These are found by nonlinear least-squares fitting of the model to the experimentally obtained data.

The indices describing glucose effectiveness *S*_*G*_ and insulin sensitivity *S*_*I*_ are derived [16] from the macroparameters.

(8) $$\begin{array}{c} S_{G}=\left(1-\alpha \right)\left(1+\beta \right)\frac{k_{21}\kappa {\left[I\right]}_{b}}{\left(k_{12}+\kappa {\left[I\right]}_{b}\right)},\\ S_{I}=\frac{k_{21}k_{12}\kappa }{{\left(k_{12}+\kappa {\left[I\right]}_{b}\right)}^{2}}. \end{array}$$

#### 2.2.3. Other Measures

The Matsuda or Composite Index of Insulin Sensitivity [18] was determined using the basal glucose and insulin data and that taken at 30, 59, 90, and 125 minutes of the oral test. Body Composition was determined using DXA (GE Lunar Prodigee).

#### 2.2.4. Statistical Analysis

Measured parameters are quoted as mean ± standard error. Derived parameter values are quoted as mean values with maximum and minimum values following in parentheses. All comparisons are made using nonparametric methods, principally Spearman correlation.

## 3. Results

Eleven volunteers (three female) were recruited for the study. The subject characteristics are shown in Table 1.

As expected, clamp-derived endogenous glucose production (EGP) in the basal state was strongly correlated with weight (*r*_*s*_ = 0.909, *P* < 0.001), with the weight normalized average of 1.94 (1.48–2.08) mg/kg/min. The mean plasma insulin concentration during the clamp phase was 139 ± 6 pmole·L^−1^. In all the subjects except for one, this level of hyperinsulinaemia was sufficient to suppress EGP by 82 ± 7%.

The *M*/*I* indices of insulin sensitivity for the subjects are presented in Table 2. This clamp index is positively correlated with Lean Body Mass (*r*_*s*_ = 0.782, *P* < 0.01) and negatively correlated with fraction of body fat (*r*_*s*_ = −0.627, *P* < 0.05).

However, there is no significant relationship with either weight (*r*_*s*_ = 0.36) or with BMI (*r*_*s*_ = 0.16). Estimates of insulin sensitivity from the clamp and the Matsuda index were correlated (*r*_*s*_ = 0.55), although this did not quite achieve significance with this number of subjects.

The indices of glucose parameters obtained from the ODILE test are given in Table 2. In contrast to the original formulation of the two compartment model [14], the improved parameterization [15] adopted here forces plasma clearance rate to bear a fixed ratio of 1/(1 − *α*) to *S*_*G*_, and therefore the latter parameter is not reported. The mean value of glucose effectiveness was 0.172 (0.0126–0.0259) min^−1^ and that of insulin sensitivity 2.07 × 10^−4^(1.07 × 10^−4^–3.51 × 10^−4^) L·pmole·min^−1^. The two parameters were uncorrelated (*r*_*s*_ = 0.31).

Although *S*_*G*_ was not related to any body composition parameter, in contrast, there was a strong relationship between *S*_*I*_ and lean body mass (*r*_*s*_ = −0.700, *P*~0.02); the correlation of insulin sensitivity with BMI was stronger than that observed with the clamp estimate and that with percent body fat weaker, with neither achieving statistical significance for *S*_*I*_.

**Figure 2:**
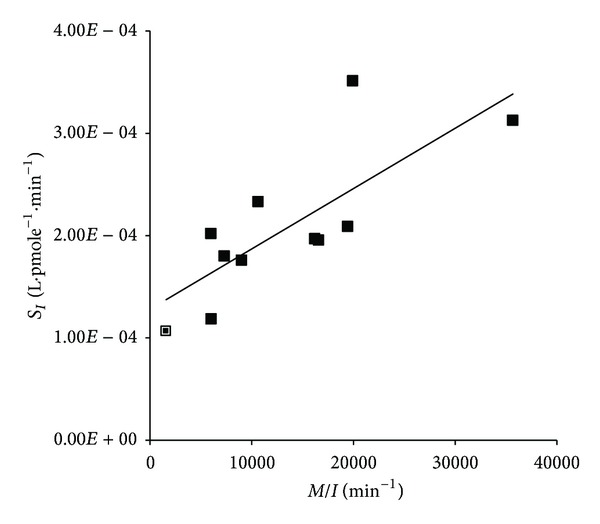
Comparison of the estimates of insulin sensitivity from the ODILE test and the clamp. The point outlined represents the subject for which EGP suppression was not achieved during the clamp.

The estimates of insulin sensitivity from the clamp and ODILE test (*M*/*I* and *S*_*I*_) were highly correlated (*r*_*s*_ = +0.764, *P* < 0.05), Figure 2. Least-squares fitting produced a line which had a significant intercept with the *S*_*I*_ axis.

## 4. Discussion

The development of tests, which provide rigorous estimates of the parameters of glucose disposal and production under normal physiological conditions, is of great interest to both clinicians and epidemiologists interested in the pathology of type 2 diabetes. However, the application and interpretation of such tests require care since insulin has two distinct modes of action. Firstly, it accelerates glucose disposal by increasing the number of plasma membrane glucose transporters. This takes place mainly in nonhepatic tissue. Secondly, insulin signals to the liver to shift the balance between the rates of glucose storage and release into the circulation in favour of the storage process. Crude models of insulin sensitivity therefore have two terms, one describing the disposal mechanisms (peripheral insulin sensitivity) and one the inhibition of endogenous glucose production (EGP) usually referred to as hepatic insulin sensitivity.

Dysfunctionality in either peripheral or hepatic insulin sensitivity will contribute to the metabolic disturbance observed in subjects with impaired glucose tolerance (IGT), diabetes mellitus (DM), or the metabolic syndrome. Although it is well-established that both mechanisms are defective in these states, the relative magnitudes of the abnormalities in specific states of disease are still a matter for discussion. Whilst many workers have concluded that the major contribution to insulin resistance in type 2 diabetes is in peripheral tissues and indeed direct measurement of the impairment of glycogen formation in skeletal muscle has been made [19], there is contrary evidence to suggest that glucose tolerance is primarily determined by hepatic insulin sensitivity [20].

Some of this ambiguity may be caused by the nature of the test used in the insulin sensitivity measurement. Many tests are performed in the postabsorptive state, where it has been shown that EGP is normal even in moderately hyperglycaemic subjects [21]. Currently, there are only two accredited methodologies that are considered to give accurate measures of insulin sensitivity [1]: the euglycaemic hyperinsulinaemic clamp and the intravenous glucose tolerance test (IVGTT) interpreted in terms of the minimal model. However, neither of these tests are made under conditions which can be regarded as truly normal physiology. Any study utilising intravenous administration of glucose or insulin can be considered to be in “reverse physiology” since the systemic concentrations of the administered substance will exceed that in the hepatic portal vein, contrary to the situation found under free-living.

Oral administration of glucose, either directly as in the oral glucose tolerance test (OGTT) or in more complex naturally occurring forms as sugars and starches in food, provides physiologically realistic conditions but introduces considerable complexity into the interpretation of the test since now the plasma glucose concentration profile after dose administration becomes the sum of three terms: absorption from the gastrointestinal tract, endogenous production, and disposal. This has led investigators to introduce various empirical methods of obtaining indices of insulin sensitivity from oral tests {Sluiter, 1976 #1405 Hanson, 2000 #1729; Cederholm, 1990 #1406; Matsuda, 1999 #1163; Belfiore, 1998 #1402}. The major difficulty with this approach is that at best the results obtained will be ordinally related. In order to quantitatively express concepts such as improvements in insulin sensitivity in response to pharmacological or dietary intervention, it is required that the data obtained should lie on an interval scale, and this is best achieved by retaining some sort of physiological model which can be rigorously described mathematically.

Recently, we demonstrated the feasibility of a different approach to obtaining values of insulin sensitivity under the physiological conditions of an oral glucose dose {Bluck, 2006 #2739}. In our method, which we since renamed the Oral Dose Intravenous Label Experiment (ODILE), the strategy adopted is effective to separate the hot and cold glucose doses given in the hot IVGTT. As in the hot IVGTT [22, 23], the labelled species are used for the determination of the metabolic parameters; the associated cold glucose merely provides the insulin secretory response.

In our new approach, although the major fraction of the glucose dose is given orally, intravenous administration of the label was required to circumvent modeling the absorption characteristics. Secondly, the quantity of labeled material had to be small enough not to perturb the extant kinetics of glucose clearance. Whilst this would have been a simple matter with radiolabel, the quantity of ethically acceptable deuterated glucose given would have been too great. This led us to consider changing the method of detection from gas-chromatography/mass spectrometry (GC/MS) to the more precise gas chromatography/combustion/mass spectrometry (GC/C/MS). Recently, we demonstrated that [1]-^13^C-glucose was a satisfactory alternative to the customary [6,6]-^2^H_2_-labeled material in the IVGTT for the determination of glucose metabolism [12, 13] in a wide variety of states of glucose tolerance [24]. Compound specific ^13^C isotope ratio analysis is a well-established technique [25–27] and we were able to apply it in our desired application using only 250 mg of labeled material.

Our preliminary investigation [8] proved the principle of the new methodology, indicating that for intravenous glucose tolerance tests using the orally stimulated (OSIVGTT) protocol interpretation using the 2CMM were not only desirable but necessary. In addition, we showed that the values obtained for parameters of glucose metabolism from the OSIVGTT seemed to be greater than those from the corresponding IVGTT by a factor of about 2.5.

Although it can be regarded as nonphysiological, and despite its time-consuming and labor intensive nature, the euglycaemic hyperinsulinaemic euglycaemic clamp [2] is still regarded as the method of choice by diabetologists for the assessment of insulin sensitivity. This is principally because it is easily interpreted, does not require a model of the glucose and insulin system, and depends on very few assumptions. For this reason, any new methodology for estimating insulin sensitivity is usually validated by the comparison with clamp techniques, and this is the purpose of this report for the ODILE method.

The significant correlation achieved between the clamp and ODILE method demonstrates that the latter is a valid tool for the assessment of insulin dependent glucose disposal; this has been achieved with such a relatively homogenous group of subjects is particularly encouraging. However, it is of some concern that the two methods are apparently not measuring the same metabolic parameter, since the intercept of the plot, Figure 2, is clearly not equal to zero. This parallels closely the situation for the IVGTT itself, which has been compared with clamps on a number of occasions [3, 7, 28–31]. A significant relationship between the IVGTT and clamp was not achieved in an early study with a very homogenous population [29], but was later demonstrated when wider spectrums of insulin sensitivity were investigated [3, 7].

In this context, it should be noted that the relationship between the clamp and ODILE measures of insulin sensitivity remains even when corrected for lean body mass, although it is slightly weaker (data not shown). This is important as although both measures are themselves correlated with lean body mass, the observed association is not simply due to cross correlation.

As in the present investigation, it has been pointed out that the IVGTT and clamp are correlated, but not measuring the same metabolic parameter [30]. In the case of the ODILE test this is perhaps not surprising, given that, unlike in the clamp, where measurements are made under steady conditions, in the ODILE test plasma insulin changes in a continuous manner in response to the pattern of meal-induced hyperglycaemia, making it likely that the partitioning of the glucose load between the various metabolic routes is different for the clamp and ODILE in the clamp the insulin infusion elevated plasma levels to about 140 pmole/L; in the OSIVGTT this value was exceeded from about 0.5 hr before the administration of the tracer to 1.5 hr afterwards and reached a maximum value of 330 pmole/L. During this period, the dose-response curve for insulin mediated glucose uptake is near-saturated in splanchnic tissue but not in muscular organs such as the leg [32], and therefore it might be expected that insulin sensitivity would be a function of the plasma insulin profile during the measurement period. Secondly, ODILE, unlike the clamp or IVGTT, operates in circumstances of normal physiology where the concentrations of both glucose and insulin are higher in the hepatic portal vein than in the peripheral systemic circulation.

Under the conditions of ODILE endogenous, insulin is secreted into the hepatic portal system, and encounters the liver, where approximately half of the hormone is degraded, before passing to the peripheral tissues. Elevated portal insulin has been shown to stimulate glycogenesis by a direct route, but insulin administered intravenously as in the clamp, acts primarily by stimulating gluconeogenesis, and thereby increasing glycogenesis by an indirect route [33]. An effect of the origin of insulin on estimates of insulin sensitivity derived from the minimal model has been demonstrated by comparison of tolbutamide-modified and insulin-modified tests [34].

Similarly, it has long been known that the route of administration of glucose has a profound effect on its metabolism [35]. Under the initial conditions of oral glucose ingestion, the concentration of glucose in the portal system exceeds that in the peripheral veins, and animal models indicate that this portal signal is sufficient to transform liver metabolism from net glucose production to net storage [36].

## 5. Conclusions

The ODILE test was invented specifically to apply the power of tracer methodologies under truly physiologically realistic conditions. In order to increase its acceptability to the diabetological community, we have validated the test against a “gold standard” methodology and demonstrated that, whilst the tests are not directly comparable, the correlation between them, even in a relatively homogenous population, is adequate and better than that obtained from an established interpretation of the oral glucose tolerance test (Matsuda index). Furthermore, the ODILE test requires considerable less clinical expertise than the clamp and is more suited to medium scale studies of glucose metabolism.

## Figures and Tables

**Table 1:** Subject characteristics. Fat mass and Lean mass (kg) obtained from DXA measurement.

ID	Sex	Age (y)	Height (m)	Weight (kg)	Fat mass	Lean mass
A	F	31	1.58	50.5	14.5	34.2
B	M	22	1.84	79.5	11.4	64.5
C	M	24	1.76	70.5	4.1	62.9
D	M	21	1.80	91.1	20.0	67.6
E	F	22	1.65	55.8	13.8	39.9
F	F	23	1.65	62.2	20.9	38.5
G	M	23	1.78	86.2	24.9	57.6
H	M	20	1.79	75.0	19.8	52.1
I	M	21	1.78	80.0	25.5	51.7
J	M	28	1.75	88.2	37.8	47.9
K	M	22	1.86	81.1	6.9	70.9

**Table 2:** Parameters of glucose metabolism derived from the clamp and the OSIVGTT.

ID	*M/I* (min^−1^)	*S*_*G*_ (min^−1^)	*S*_*I*_ (L·pmole·min^−1^)
A	9001	0.0259	1.76 × 10^−4^
B	16567	0.0126	1.96 × 10^−4^
C	16197	0.0138	1.97 × 10^−4^
D	35674	0.0215	3.13 × 10^−4^
E	1584	0.0201	1.07 × 10^−4^
F	5996	0.0126	1.19 × 10^−4^
G	7305	0.0149	1.80 × 10^−4^
H	19438	0.0151	2.09 × 10^−4^
I	10627	0.0168	2.33 × 10^−4^
J	5986	0.0143	2.02 × 10^−4^
K	19920	0.0220	3.51 × 10^−4^

## References

[1] Monzillo LU, Hamdy O (2003). Evaluation of insulin sensitivity in clinical practice and in research settings. *Nutrition Reviews*.

[2] de Fronzo RA, Tobin JD, Andres R (1979). Glucose clamp technique: a method for quantifying insulin secretion and resistance. *The American Journal of Physiology-Endocrinology Metabolism and Gastrointestinal Physiology*.

[3] Bergman RN, Ider YZ, Bowden CR, Cobelli C (1979). Quantitative estimation of insulin sensitivity. *The American Journal of Physiology-Endocrinology Metabolism and Gastrointestinal Physiology*.

[4] Wagenknecht LE, Mayer EJ, Rewers M (1995). The insulin resistance atherosclerosis study (iris): objectives, design, and recruitment results. *Annals of Epidemiology*.

[5] Jebb SA, Frost G, Griffin B (2007). The RISCK study: testing the impact of the amount and type of dietary fat and carbohydrate on metabolic risk. *Nutrition Bulletin*.

[6] Groop LC, Widen E, Ferrannini E (1993). Insulin resistance and insulin deficiency in the pathogenesis of type 2 (non-insulin-dependent) diabetes mellitus: errors of metabolism or of methods?. *Diabetologia*.

[7] Saad MF, Anderson RL, Laws A (1994). A comparison between the minimal model and the glucose clamp in the assessment of insulin sensitivity across the spectrum of glucose tolerance. *Diabetes*.

[8] Bluck LJC, Clapperton AT, Coward WA (2006). A stable isotope minimal model protocol with oral glucose administration. *Rapid Communications in Mass Spectrometry*.

[9] Jackson SJ, Waterhouse JS, Bluck LJC (2007). A single glucose derivative suitable for gas chromatography/mass spectrometry and gas chromatography/combustion/isotope ratio mass spectrometry. *Rapid Communications in Mass Spectrometry*.

[10] Bluck LJC, Coward WA (1997). Peak measurement in gas chromatographic mass spectrometric isotope studies. *Journal of Mass Spectrometry*.

[11] Bluck LJC, Coward WA (2004). The application of a simple algorithm to isotope ratio measurements by gas chromatography/combustion/isotope ratio mass spectrometry. *Measurement Science and Technology*.

[12] Clapperton AT, Bluck LJ (2001). Measuring insulin sensitivity using 13C glucose and gas chromatography/combustion/isotope ratio mass spectrometry. *Proceedings of the Nutrition Society*.

[13] Clapperton AT, Coward WA, Bluck LJC (2002). Measurement of insulin sensitivity indices using 13C-labelled glucose and gas chromatography/combustion/isotope ratio mass spectrometry. *Rapid Communications in Mass Spectrometry*.

[14] Caumo A, Cobelli C (1993). Hepatic glucose production during the labeled IVGTT: estimation by deconvolution with a new minimal model. *The American Journal of Physiology-Endocrinology and Metabolism*.

[15] Toffolo G, Cobelli C (2003). The hot IVGTT two-compartment minimal model: an improved version. *The American Journal of Physiology-Endocrinology and Metabolism*.

[16] Vicini P, Caumo A, Cobelli C (1997). The hot IVGTT two-compartment minimal model: indexes of glucose effectiveness and insulin sensitivity. *The American Journal of Physiology-Endocrinology and Metabolism*.

[17] Mari A (1997). Assessment of insulin sensitivity with minimal model: role of model assumptions. *The American Journal of Physiology-Endocrinology and Metabolism*.

[18] Matsuda M, de Fronzo RA (1999). Insulin sensitivity indices obtained from oral glucose tolerance testing: comparison with the euglycemic insulin clamp. *Diabetes Care*.

[19] Shulman GI, Rothman DL, Jue T, Stein P, de Fronzo RA, Shulman RG (1990). Quantitation of muscle glycogen synthesis in normal subjects and subjects with non-insulin-dependent diabetes by 13C nuclear magnetic resonance spectroscopy. *The New England Journal of Medicine*.

[20] Båvenholm PN, Pigon J, Östenson C-G, Efendic S (2001). Insulin sensitivity of suppression of endogenous glucose production is the single most important determinant of glucose tolerance. *Diabetes*.

[21] de Fronzo RA, Ferrannini E, Simonson DC (1989). Fasting hyperglycemia in non-insulin-dependent diabetes mellitus: contributions of excessive hepatic glucose production and impaired tissue glucose uptake. *Metabolism*.

[22] Avogaro A, Bristow JD, Bier DM, Cobelli C, Toffolo G (1989). Stable-label intravenous glucose tolerance test minimal model. *Diabetes*.

[23] Cobelli C, Pacini G, Toffolo G, Sacca L (1986). Estimation of insulin sensitivity and glucose clearance from minimal model: new insights from labeled IVGTT. *The American Journal of Physiology-Endocrinology and Metabolism*.

[24] Bluck LJC, Clapperton AT, Coward WA (2005). 13C and 2H-labelled glucose compared for minimal model estimates of glucose metabolism in man. *Clinical Science*.

[25] Douthitt CB (1999). Hyphenation of gas chromatographic techniques with isotope ratio mass spectrometry: present status and future. *Analusis*.

[26] Meier-Augenstein W (1999). Applied gas chromatography coupled to isotope ratio mass spectrometry. *Journal of Chromatography A*.

[27] Midwood AJ, McGaw BA (1999). Recent developments in the analysis of light isotopes by continuous flow isotope ratio mass spectrometry. *Analytical Communications*.

[28] Beard JC, Bergman RN, Ward WK, Porte D (1986). The insulin sensitivity index in nondiabetic man: correlation between clamp-derived and IVGTT-derived values. *Diabetes*.

[29] Donner CC, Fraze E, Chen Y-DI (1985). Presentation of a new method for specific measurement of in vivo insulin-stimulated glucose disposal in humans: comparison of this approach with the insulin clamp and minimal model techniques. *Journal of Clinical Endocrinology & Metabolism*.

[30] Foley JE, Chen YDI, Lardinois CK (1985). Estimates of in vivo insulin action in humans: comparison of the insulin clamp and the minimal model techniques. *Hormone and Metabolic Research*.

[31] Swan JW, Walton C, Godsland IF (1994). Assessment of insulin sensitivity in man-a comparison of minimal model-derived and euglycemic clamp-derived measures in health and heart-failure. *Clinical Science*.

[32] Basu R, Basu A, Johnson CM, Schwenk WF, Rizza RA (2004). Insulin dose-response curves for stimulation of splanchnic glucose uptake and suppression of endogenous glucose production differ in nondiabetic humans and are abnormal in people with type 2 diabetes. *Diabetes*.

[33] Satake S, Moore MC, Igawa K (2002). Direct and indirect effects of insulin on glucose uptake and storage by the liver. *Diabetes*.

[34] Kiser KM, Prigeon RL, Kahn SE (2003). Insulin sensitivity quantified with the minimal model is lower with the insulin-modified than the tolbutamide-modified frequently sampled intravenous glucose tolerance test. *Journal of Investigative Medicine*.

[35] de Fronzo RA, Ferrannini E, Hendler R (1978). Influence of hyperinsulinemia, hyperglycemia, and the route of glucose administration on splanchnic glucose exchange. *Proceedings of the National Academy of Sciences of the United States of America*.

[36] Pagliassotti MJ, Holste LC, Moore MC, Neal DW, Cherrington AD (1996). Comparison of the time courses of insulin and the portal signal on hepatic glucose and glycogen metabolism in the conscious dog. *Journal of Clinical Investigation*.

